# Biomineralization for Reducing and Controlling Sand‐Dust Storms

**DOI:** 10.1002/advs.202403961

**Published:** 2024-06-26

**Authors:** Linchang Miao, Hengxing Wang, Xiaohao Sun, Linyu Wu

**Affiliations:** ^1^ Institute of Geotechnical Engineering Southeast University Nanjing Jiangsu 210096 China

**Keywords:** biomineralization, sand‐dust storms, sand‐dust weather, water storage capacity

## Abstract

The sand‐dust weather and sand‐dust storms have become a serious environmental disaster worldwide. It is an important challenge to develop technologies for desert sand solidification in order to prevent and control sand‐dust weather. The biomineralization technology for solidifying desert sands has been a novel method for reinforced soils in recent years. The biomineralization solidification sand field tests are completed at the Wuma Highway solidification section in the Tengger Desert. The superiority of the biomineralization for solidifying sands is verified by measuring the water storage capacity of different reinforcement zones including bare sand zone, plant zone, biomineralization solidifying sand zone, and biomineralization combined plant solidifying sand zone. Simultaneously, the molecular dynamics calculation analysis is used to verify the role of biomineralization solidifying sands in preventing sand‐dust storms. All results demonstrate that the biomineralization solidification sand method is effective for controlling and preventing sandstorm disasters.

## Graphical abstract

The sand‐dust weather and sand‐dust storms have become a serious environmental disaster worldwide. The biomineralization technology for solidifying desert sands is a novel method for reinforced soils in recent years. The superiority of the biomineralization for solidifying sands is verified by the field tests completed in the Tengger Desert and the molecular dynamics calculation analysis.

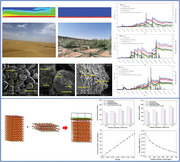

## 1 Introduction

Asia is the second‐largest dust source in the world after Africa. There are extensive deserts distributed across Kazakhstan, northwest China, Mongolia, and India.^[^ ^1^, ^2^ ^]^ A large amount of dust from these deserts is emitted into the overlying atmosphere and transported to downstream areas. Statistics indicate that ≈600 Tg of East Asian dust is released into the atmosphere annually from the Taklimakan Desert and Gobi Desert.^[^ ^3^ ^]^ This dust can sweep across the whole of northern China^[^ ^4^, ^5^ ^]^ and a large portion of eastern China,^[^ ^6^ ^]^ affecting regional and global weather and climate.^[^ ^7^, ^8^ ^]^ The Central Asia dust belt, characterized by an arid climate, extends from the Caspian Sea to the East Tianshan Mountains in the eastern end of Central Asia. The dust‐sand storms can bring ecological and biogeochemical benefits, such as nutrient transport; however, the frequently occurring and rather intensive sand‐dust storms in the dust belt are estimated to contribute to ≈20% of global dust emissions.^[^ ^9^, ^10^, ^11^, ^12^, ^13^, ^14^, ^15^, ^16^, ^17^, ^18^ ^]^ These sand‐dust storms have attracted broad scientific interest due to their influences on socioeconomics, human health, and ecosystems,^[^ ^10^, ^11^, ^12^, ^15^, ^18^, ^19^ ^]^ particularly motivating numerous studies on dust activity and its associated land and atmospheric characteristics and dynamics.^[^ ^16^, ^20^, ^21^ ^]^

To improve the air quality in China, the Chinese government promulgated the Action Plan for Air Pollution Prevention and Control in 2013. The plan clearly stated that by 2017, the concentration of particular matter (PM_2.5_) at the city level and above would decrease by 10% compared to that in 2012, and the number of good air quality days would increase year by year. The Action Plan played a significant role in reducing sand‐dust storms from 2013 to 2020. However, the outbreak of sand‐dust storms in northern China from 2021 to 2023 has become a serious environmental issue, garnering considerable attention from the scientific community. Addressing the challenge of eradicating dust and sandstorms is an imperative task. The key to solving this problem lies in preventing the sand‐dust emissions driven by the wind.

The area with severe desertification contains an abundance of aeolian sandy soils without strong cohesion, which are readily transported over vast distances by wind. Dust‐sand storms as a common meteorological phenomenon easily occur due to climate change and land degradation;^[^ ^22^ ^]^ consequently, it is urgent to control sandstorms. To date, there are three prevailing methods for sandstorm control: engineering, vegetation, and chemical methods.^[^ ^23^, ^24^ ^]^ The engineering method is the most widely applied, but it has a limited service life. The vegetation approach always has a low plant survival rate. In the case of chemical methods, several chemical materials may pose adverse effects on the ecosystem.^[^ ^25^ ^]^ In response to these limitations, novel biochemical strategies such as microbially induced calcite precipitation (MICP) or enzymatically induced calcite precipitation (EICP) as a biomineralization technique have emerged with promising potential to solidify desert sands and control sandstorms.^[^ ^26^, ^27^, ^28^, ^29^, ^30^ ^]^ Therefore, the biomineralization method is proposed as the most effective method for preventing and controlling sand‐dust storms in the dust belt, which was also utilized in this study for field dust and storm control. However, research on the field application of biomineralization for sandstorm control and related mechanisms of molecular dynamics remains in its infancy; therefore, field testing was conducted in this study, and calculation was combined for an in‐depth investigation.

### 1.1 Method and Calculation of Biomineralization Solidifying Sands

#### 1.1.1 Biomineralization Method

The biomineralization method provides an alternative method for sandstorm control. For the biomineralization method, the urease enzymes can hydrolyze urea (CO(NH_2_)_2_) into carbonate ions (CO_3_^2−^) and ammonium (NH_4_^+^), as described by Equation (1). The resulting carbonate ions bind with calcium ions (Ca^2+^) supplied by a calcium acetate solution (Ca(CH_3_COO)_2_) to produce carbonate precipitate, such as calcium carbonate (CaCO_3_), as shown in Equation (2).

(1) $$\mathrm{CO}{\left(NH_{2}\right)}_{2}+2H_{2}O\to 2N{H_{4}}^{+}+C{O_{3}}^{2-}$$

(2) $$C{O_{3}}^{2-}+\;Ca^{2+}\;\to \mathrm{CaC}O_{3}$$

The content of calcium carbonate in biomineralization depends on urea hydrolysis rate. The urea hydrolysis rate according to the Michaelis–Menten kinetics equation can be used to determine the consumption rate for diluted chemical species (i.e., urea, calcium, and ammonium):

(3) $$r_{ureolysis}=C_{urease}\;k_{u}\frac{C_{urea}}{k_{m,\;urea}+C_{urea}}\exp\left(-\frac{C_{Ca^{2+}}}{k_{iCa}}\right)$$

where *C_urease_* is the urease concentration *k_u_* is the specific ureolysis rate (mol/(L·s·OD)); *C_urea_* is the urea concentration; *k*_*m*,*urea*_ is the half‐saturation constant (mol L^−1^) for urea; $C_{Ca^{2+}}$ is the calcium source concentration (mol L^−1^); *k_iCa_* is the calcium ion concentration.

The biomineralization solidification sand field tests were completed in the Tengger Desert in August 2020, near the city of Zhongwei, Ningxia, China. The test area is located in a quicksand corridor and partial straw checkerboard barriers have been covered by aeolian sand.

During biomineralization, the first task is to obtain carbonate and calcium ions. The carbonate ions are obtained by hydrolysis of urea, while the calcium ions are supplied by a calcium acetate solution. According to the principle of biomineralization (Equations 1 and 2) and the preliminary experiments (Equation 3), the research scheme is determined. Therefore, biomineralization method (EICP) was used to solidify sands, where urea is added to a solution of plant‐derived urease enzyme with a concentration of 4000 U L^−1^ (1U corresponds to the amount of enzyme that hydrolyses 1 µmoL of urea per minute at pH 7.0 and 25 °C). The solution of plant‐derived urease enzyme was obtained by soaking 100 g of soybean powder (purchased from Shandong Wobainian Company) in 1 L of water for 2 h. Over 48 h, the urea is hydrolyzed by the urease. After hydrolyzing the urea, the resulting solution is mixed with calcium acetate solution. The concentrations of calcium ions in calcium acetate and urea in the biochemical solution are equal (0.25 m). The biomineralization solidification sand method utilized a mixed solution (0.8 L m^−2^ of urease solution and 1.2 L m^−2^ of cementation solution), which was uniformly sprinkled onto the sand surface using pumps and sprayers to form the crust layer in August 2020.

#### 1.1.2 Calculation Model of Aerodynamics

**Figure 1:**
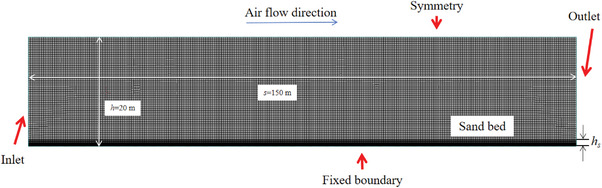
Model of aerodynamics.

Fluent software is used to construct an Euler dual fluid module to simulate the erosion of sand particles by airflow. The model of aerodynamics is a rectangular area of 150m x 20 m, with a bottom sand bed thickness of *h_s_* = 1 m, as shown in **Figure** **1**.

In the Euler model, the sand phase is set as the particle phase, with a particle size of 0.0001m. The Lun model is used for the particle volume coefficient, and the maximum stacking rate is 0.65. The Gidaspow model was chosen for both the momentum transfer coefficient model and the dynamic coefficient model. Sand particle density *ρ_s_* = 2650 kg m^−3^, and air density *ρ_g_* = 1.255 kg m^−3^. The model of biomineralization solidification is simulated by increasing dynamic viscosity, and the dynamic viscosity is 100 Pa·s.

In the section of boundary condition, the outflow is used in the outlet. Symmetry is used in the upper boundary. In the lower boundary, the air phase is a non‐slip fixed wall, and the sand phase is a partial slip boundary condition, with a mirror coefficient of 0.01. For inlet, The rough characteristics of the sand surface will affect the airflow velocity near the ground, so the airflow velocity will change with height. Therefore, the inlet adopts a fully developed atmospheric turbulent boundary layer wind speed on a stable bed surface, and the initial wind speed follows a logarithmic vertical distribution with height direction. The velocity profile follows the logarithmic distribution pattern of the von Karman boundary layer, as shown in Equation (4).

(4) $$u_{z}=\frac{u^{*}}{k}\ln\left(\frac{z-d}{z_{0}}\right)$$

where *u_z_* is the wind speed (m ^−1^s) at height *z* (m), u^*^ is the wind speed (m ^−1^s) at height z (m), *k* is the von Karman constant (usually taken as 0.4), *z*_0_ is the surface roughness (m), usually taken as *d_s_*/30, *d_s_* is the diameter of sand particles; *z* is the height (m); *d* is the zero plane position (m).

#### 1.1.3 Calculation Method of Molecular Dynamics

The method of biomineralization solidifying sands is to form the hardened layers on the desert surface through calcium carbonate cementation to prevent and control sand‐dust storms during biomineralization in the dust belt. The permeability coefficient and porosity of the desert sands after biomineralization solidifying sands will be decreased as the calcium carbonate cementation. Therefore, the molecular dynamics can be used to calculate the growth model of calcium carbonate precipitation, the cementation work of calcium carbonate, and state parameters of desert sands for the biomineralization of solidifying sands.

The transport equation governing the transmission flow of biochemical solution in the biomineralization sand‐fixation process is described by Darcy's equation as follows:

(5) $$\frac{\partial }{\partial t}\left(\rho n\right)+\nabla \cdot (\rho u)\;=Q_{m}\;$$

where *n* is the porosity of the specimen, *Q_m_* is the quality source term expressed in kg m^−3^s^−1^, and *ρ* is the density expressed in kg m^−3^.

The diffusion equation of the biochemical solution in the sand‐fixation process is expressed as follows:

(6) $$\frac{\partial nC_{i}}{\partial t}=\;\nabla \left(\mathrm{nD}\cdot \nabla C_{i}\right)-\nabla \left(uC_{i}\right)+nm_{i}r$$

where *C_i_* is the concentration of component i, expressed in imol L^−1^; D is the dispersion coefficient, expressed in m^2^s^−1^, m_i_ is the contribution constant of a chemical reaction for component i, and r is the reaction speed.

The growth equation of calcium carbonate in the process of biomineralization solidifying sands is represented as:

(7) $$\frac{\partial C_{CaCO_{3}}}{\partial t}=m_{CaCO_{3}}\;nr_{p}$$

where $C_{CaCO_{3}}$ is the contents of calcium carbonate produced, expressed in kg m^−3^, and $m_{CaCO_{3}}$ is the molar mass of calcium carbonate, expressed in kg mol.

The porosity differential equation is given by:

(8) $$\frac{\partial n}{\partial t}=\frac{1}{\rho_{CaCO_{3}}}\;\frac{\partial C_{CaCO_{3}}}{\partial t}$$

where $\rho_{CaCO_{3}}$ is the calcium carbonate density, expressed in kg m^−3^.

The permeability equation is given by:

(9) $$k=k_{0}\;\frac{{\left(1-n_{0}\right)}^{2}}{{\left(1-n\right)}^{2}}{\left(\frac{n}{n_{0}}\right)}^{3}$$

where *k*_0_ is the initial permeability in m^2^, and *n*_0_ is the initial porosity. The permeability coefficient can be obtained by converting permeability as follows:

(10) $$K\;=\;k\frac{\rho g}{\eta }$$

where *K* is the permeability coefficient in m s^−1^, and *g* is the acceleration due to gravity, ≈9.8 m s^−2^.

The molecular dynamics model for calculating the binding energy of calcium carbonate and sand is given by:

(11) $$\begin{array}{ccc} E_{total} & = & \sum_{bond}E_{b}\left(b\right)+\sum_{angle}E_{\theta }\left(\theta \right)+\sum_{dihedral}E_{\phi }\left(\phi \right)\\ & & +\sum_{out-of-plane}E_{\chi }\left(\chi \right)+\sum_{cross}E\left(b,\theta ,\phi \right)+E_{coulomb}+E_{vdw} \end{array}$$

where *E_total_* is the total potential energy of the system, $\sum_{bond}E_{b}\left(b\right)$ is the bond length potential energy function, $\sum_{angle}E_{\theta }\left(\theta \right)$ is bond angle potential energy function, $\sum_{dihedral}E_{\phi }\left(\phi \right)$ is the dihedral angular potential energy function, $\sum_{out-of-plane}E_{\chi }\left(\chi \right)$ is the from‐plane vibration angle potential energy function, $\sum_{cross}E\left(b,\theta ,\phi \right)$ is the cross‐term potential energy function, *E_coulomb_* is the electrostatic interaction energy, expressed in kcal mol^−1^, and *E_vdw_* is the van der Waals interaction energy in kcal mol^−1^.

Due to silica being the main component of sandy soil, it is assumed that the interaction between calcium carbonate produced by biomineralization and the interface of sandy soil can be explained by studying the atomic scale interface between calcium carbonate and silica. Materials studio software was used to construct a CaCO_3_/SiO_2_ interface model. The interaction energy and adhesion work between calcite and silica were calculated to reveal the micro mechanism of EICP improving sand properties.

SiO_2_ and CaCO_3_ crystal cells were introduced from the MS database, and their cell constants are shown in **Table** **1**.

**Table 1:** Cell constant of SiO2 and CaCO3.

Crystal cell	Edge length [Å]			Angle [°]		
	*a*	*b*	*c*	*α*	*Β*	*γ*
SiO2	4.909996	4.909996	5.402	90	90	120
CaCO3	4.990001	4.990001	17.061	90	90	120

**Figure 2:**
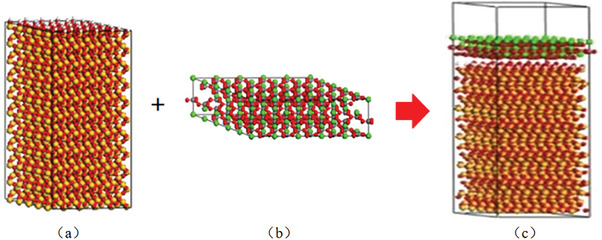
Molecular dynamics calculation models a)Sand grains contact model (SiO_2_ model), b) CaCO_3_ model, c) Model of calcium carbonate bonding with sand grains).

The stable crystal was cut plane in the [0,0,1] direction with a thickness of 10.333 Å. The crystal cells were expanded by 5 times in the x and y directions, and a vacuum layer was added to form SiO_2_ to CaCO_3_ with 2D periodic boundary conditions, shown in **Figure** **2**a,b. The SiO_2_ model and CaCO_3_ models with different particle numbers were optimized using geometric optimization, with an energy convergence level of 1 × 10^−4^ kcal mol^−1^. Place the CaCO_3_ layer on top of the hydrated SiO_2_ layer, and establish a 30 Å vacuum layer above the CaCO_3_ layer to avoid mirror interactions along the z‐direction. The interface model of CaCO_3_/SiO_2_ is shown in Figure 2c.

## 2 Test Results

**Figure 3:**
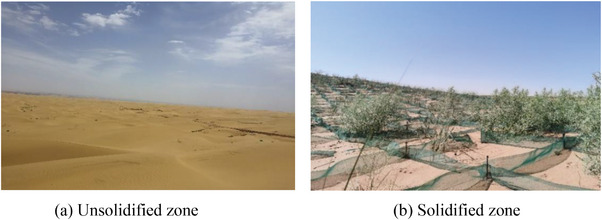
Comparison of results a) before and b) after biomineralization solidification sands in the desert field in August 2023.

**Figure 4:**
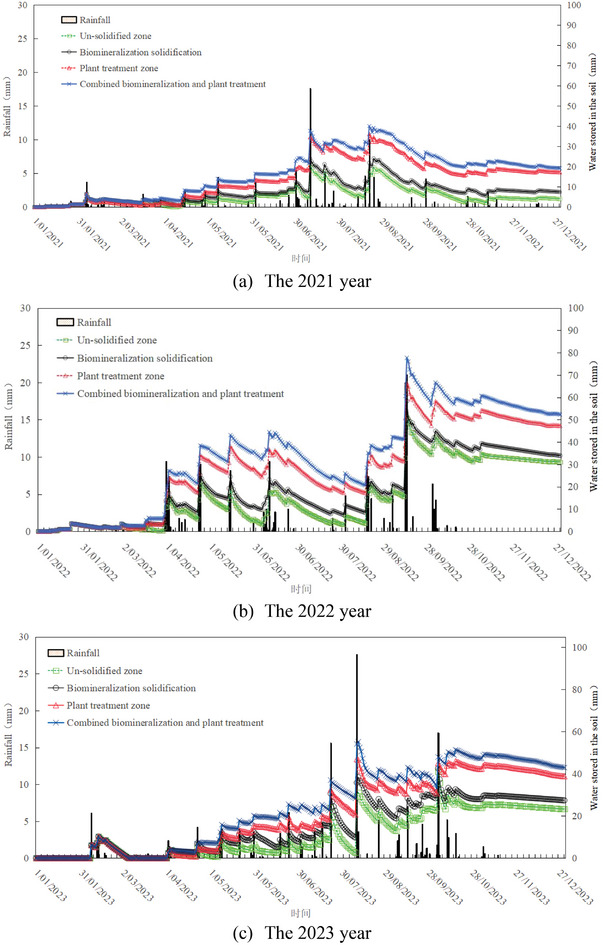
Measurement results of water storage capacity for different zones in the desert fields.

**Figure** **3** shows the effect of biomineralization solidification in the field, with photos taken in August 2023. According to the field observation, the survival rate of plants in the un‐solidified zone was below 10%; however, the survival rate of plants was over 60% in the biomineralization solidification sand zone of the desert field. It serves as the most effective illustration of the impact of biomineralization solidification sand. The biomineralization method and the biomineralization combined plant method can enhance the water storage capacity of sandy soils. **Figure** **4** presents the measurement results of water storage capacity for different zones in the field. Compared with the un‐solidified zone, biomineralization solidification significantly enhanced the water storage capacity, particularly in the zone subjected to combined biomineralization and plant treatment. Over time, the difference in water storage capacity further increased.

**Figure 5:**
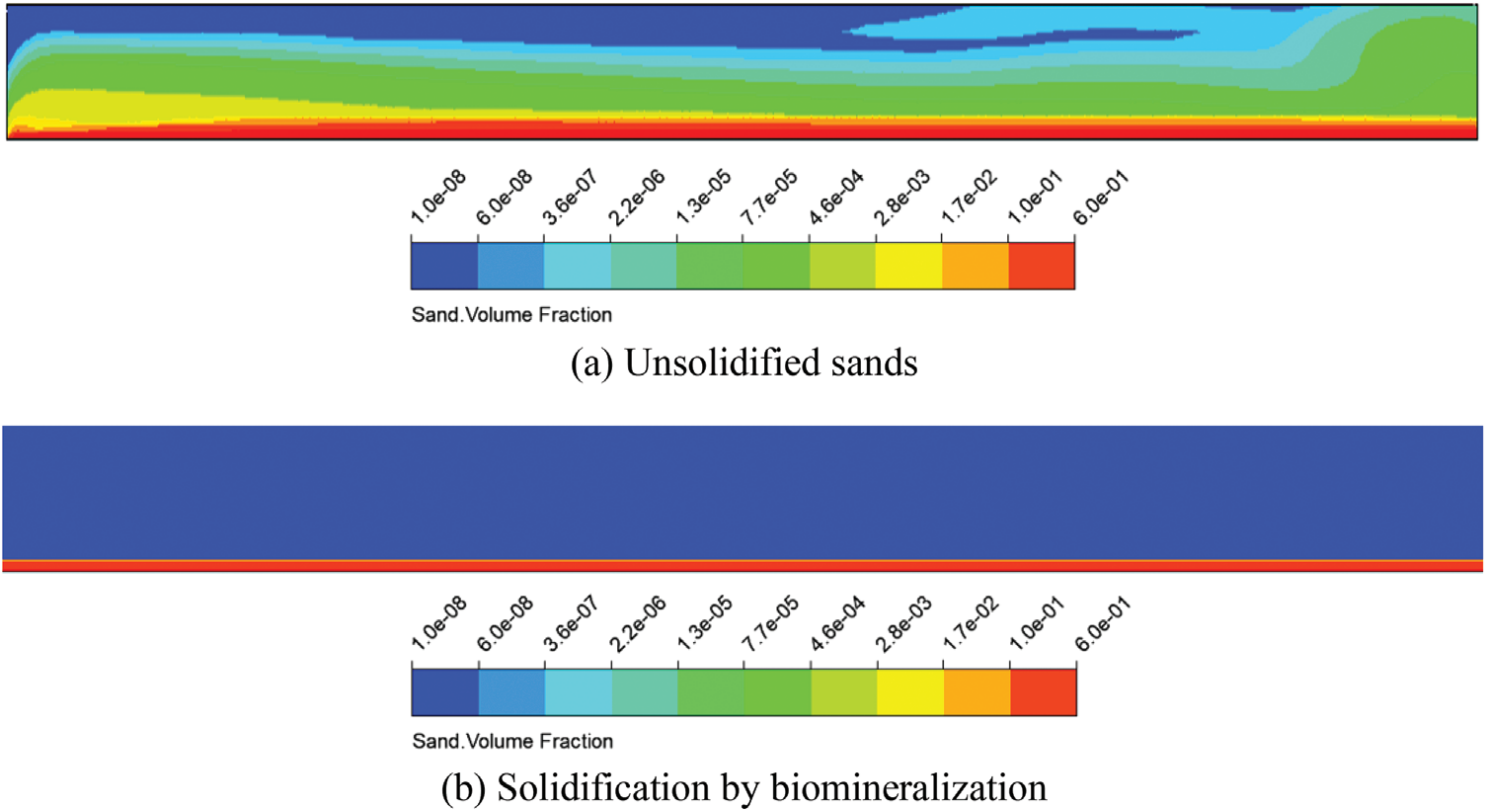
Calculation results of windbreak and sand‐fixation functions for both the biomineralization and the bare desert modes.

**Figure 6:**
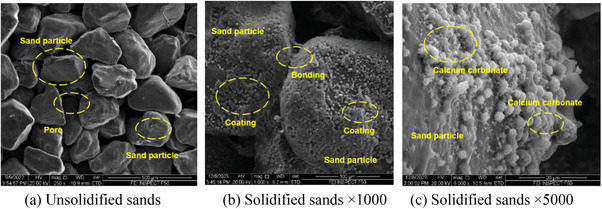
SEM images of specimens solidified by biomineralization method.

**Figure** **5** shows the calculation results of the windbreak and sand‐fixation function for both the biomineralization and the bare desert modes. According to the calculation results, minimal changes were observed in the biomineralization‐solidifying desert zone compared to the un‐solidified zone, indicating that biomineralization effectively contributes to windbreak and sand fixation. The phenomenon occurs because the surface sand particles become cemented together, forming a solidified layer through biomineralization. The scanning electron microscopy (SEM) test helps understand the cause. **Figure** **6**a depicts unsolidified sands, with pores visible between sand grains. Figure 6b,c depict the biomineralization of solidified sands at enlargements of 1000 and 5000, respectively. The pores between sand grains of biomineralization solidified sands are filled with calcium carbonate (CaCO_3_), resulting in an obvious change in pore structure. This indicates that the calcium carbonate of the biomineralization process can bond sand grains together, forming a hardened layer. This hardened layer can impede airflow when air moves. Therefore, the solidified layer created by the biomineralization method can prevent sand dust from shifting and flying, thus controlling the occurrence of sand‐dust storms. Moreover, the calcium carbonate resulting from the biomineralization changes the pore structure of desert sands and improves the water‐holding capacity of desert sands of biomineralization solidifying zone and biomineralization + plant solidifying zone in Figure 4. The results demonstrate that biomineralization solidifying desert sands can promote favorable conditions for plant growth. The method of biomineralization solidifying desert sands can effectively control and prevent the disaster of sand‐dust storms in desert sands.

## 3 Discussion

Figure 4 demonstrates the effective water storage capacity and solidification sand effects of the biomineralization combined plant system, which enhances the surface strength of the solidified layer to facilitate the optimal growth of plants. Additionally, the biomineralization solidified layer on the desert sand can prevent sand grains from being dispersed and carried by the wind. The plant growth pattern in Figure 3b has demonstrated the effect of biomineralization on sand fixation. Compared to engineering methods, biomineralization for sand fixation offers a longer service life and better adaptability to changing terrains in mobile sand areas.^[^ ^23^, ^31^ ^]^ Furthermore, the biomineralization method enhances plant survival rates, compensating for the limitations of vegetation‐based approaches. Additionally, this biochemical method is more environmentally friendly than chemical materials and promotes ecosystem restoration.^[^ ^32^ ^]^ Field results have demonstrated that the proposed biomineralization method effectively fixes sand by forming a crust layer through the application of biochemical solutions. Aiming at different sand types and environmental conditions, the treatment effect depends on the urea hydrolysis rate; according to Equation (3), the concentrations of biochemical solutions can be adjusted to achieve better treatment effects. Therefore, this method exhibits adaptability to diverse desert environments.

**Figure 7:**
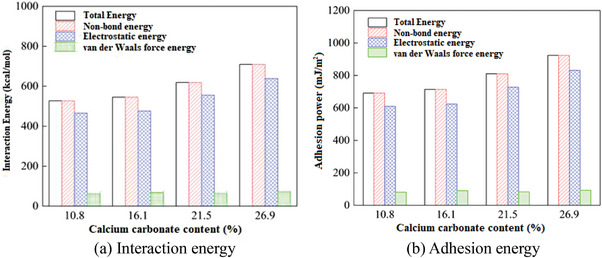
Calculation results of the a) interaction energy and b) adhesion energy.

**Figure 8:**
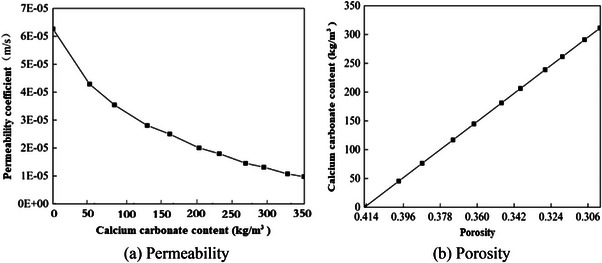
Relationship between a) permeability and b) porosity with calcium carbonate content.

The effectiveness of biomineralization solidifying sands in preventing sand‐dust storms can also be confirmed through molecular dynamics calculations. Figure 2 depicts the model of calcium carbonate cementation with sand grains to form the crust layer. Based on this model, the interaction energy between calcium carbonate and sand grains can be calculated, as well as the adhesion energy of calcium carbonate according to Equation (11). The calculation results presented in **Figure** **7** verify that both the interaction energy and the adhesion energy increase as the calcium carbonate content increases during the biomineralization solidified sand process. Different from un‐solidified loose sands, the produced calcium carbonate cemented sand particles and created a cohesive unit (crust layer), enhancing both the interaction energy and the adhesion energy, thereby impeding wind transport and improving resistance against wind erosion. Conversely, the calculation results show that the permeability coefficient and porosity decrease as the calcium carbonate content increases after the biomineralization solidification process based on Equations (8) and (9), shown in **Figure** **8**.

### 3.1 Broder Environmental Advantages of Biomineralization

In addition to sand‐dust storm control, biomineralization could potentially play a significant role in controlling the spread of anthropogenic pollution, including persistent substances like per‐ and polyfluoroalkyl substances (PFAS)^[^ ^33^, ^34^ ^]^ and microplastics.^[^ ^35^ ^]^ By stabilizing soil and reducing erosion, biomineralization may help to prevent these pollutants from being transported by wind and water, thereby mitigating their impact on ecosystems and human health.^[^ ^36^, ^37^ ^]^

### 3.2 Implication and Future Perspectives

The biomineralization method is an environmentally friendly green solidifying sand technology, which has two advantage features, one is the increasing ability to resist wind erosion due to the formed crust layer and two is increasing water retention capacity resulting from decreased permeability and porosity. Moreover, the method of biomineralization solidifying sands overcomes the deficiency of sand‐fixing engineering and can realize the goal of long‐term sand fixation. The field test results of biomineralization solidifying sands demonstrate that the survival rate of plants in the solidified area is over 60% in the biomineralization solidification sand zone of the desert field. This is mainly due to the improvement of water holding capacity after biomineralization solidifying sands. Meanwhile, the formation of the crust layer is also beneficial for nutrient retention, and the improved water‐holding capacity and nutrient retention both result in an increase in the survival rate of plants.^[^ ^38^, ^39^ ^]^ When the biomineralization method is combined with plant growth for the field sand‐dust storm control, ammonium ions can be effectively utilized and absorbed by the plants as nitrogen fertilizer. This symbiotic approach promotes plant growth without causing adverse environmental impacts. The method of biomineralization solidifying sands not only can control sand‐dust storms but also will be a new technical route of carbon sequestration. The method of biomineralization solidifying sands will become a new environmental protection technology to improve desert ecology in the near future.

## Conflict of Interest

The authors declare no conflict of interest.

## Author Contributions

L.M. was associated with methodology, experiment, investigation, funding acquisition, wrote the original draft and edited the final manuscript. H.W. performed experiment, investigation, data curation, and calculation. X.S. performed experiment, investigation, and data Curation. L.W. performed experiment, investigation, and calculation.

## Data Availability

Research data are not shared.
